# Input to the Language Learning Infant: The Impact of Other Children

**DOI:** 10.1111/desc.70045

**Published:** 2025-07-20

**Authors:** Johanna Schick, Moritz M. Daum, Sabine Stoll

**Affiliations:** ^1^ Institute for the Interdisciplinary Study of Language Evolution University of Zurich Zurich Switzerland; ^2^ Department of Psychology University of Zurich Zurich Switzerland; ^3^ Jacobs Center for Productive Youth Development University of Zurich Zurich Switzerland

## Abstract

In urban, industrialized cultures, the best predictor of how children acquire their native language is child‐directed speech from adults. However, in many societies, children are much less exposed to such input. What has remained unexplored is the impact of another type of input: other children's speech. In cross‐cultural head‐turn experiments, we demonstrate that Shipibo‐Konibo infants (Peruvian Amazon) and Swiss infants (urban industrialized setting) show greater attention to children talking among themselves than to adults doing the same. We further show that, despite hearing more child‐directed speech than child speech, Swiss infants equally attend to child‐directed speech by adults and child speech. Interestingly, child‐directed speech and child speech share acoustic and structural features. These findings suggest that, if available, the speech of other children may play an important role in language acquisition.

## 1 Introduction

Across all human cultures, children learn to speak their native language in the first years of their life. The speech addressed to them by adults (child‐directed speech) plays a unique role in this context and seems to be the best predictor for learning (e.g., Huttenlocher et al. 2010; Rowe 2012; Weisleder and Fernald 2013; Newman et al. 2016). Child‐directed speech is characterized by specific acoustic and structural features, such as higher and more variable pitch or more frequent repetitions. Infants seem to prefer child‐directed speech over adult‐directed speech in their first and second year of life (Fernald 1985; Cooper and Aslin 1990; Newman and Hussain 2006; ManyBabies 2020). Most research on child‐directed speech and its role in language development is based on data from children who grew up in Western, child‐centered cultures (Kidd and Garcia 2022). In such environments, children typically engage in dyadic interactions with adults and often receive large amounts of child‐directed speech long before they can produce their first words. However, several anthropological studies have documented a wide range of caregiver–child interactions across human cultures, providing evidence for considerable cross‐cultural variation in child‐rearing practices (e.g., Ochs and Schieffelin 1984; Gaskins 2020; Keller 2022). Within and across cultures, the amount of child‐directed and child‐surrounding input children are exposed to varies considerably (Shneidman and Goldin‐Meadow 2012; Sperry et al. 2019; Casillas et al. 2020; Bergelson et al. 2019; Orena et al. 2020; Cristia 2023). In some non‐Western cultures that are less child‐centered, children receive smaller amounts of child‐directed speech, but hear more surrounding speech during their early development (Shneidman and Goldin‐Meadow 2012; Cristia et al. 2019; Sperry et al. 2019). In comparison, smaller amounts of surrounding speech and higher amounts of directed speech have been reported for children growing up in the United States (Shneidman and Goldin‐Meadow 2012). New perspectives on language learning consider more diverse cultural environments (Correa‐Chávez and Rogoff 2009; Shneidman et al. 2009; Sperry et al. 2019) and growing evidence suggests that children can also learn from surrounding speech (Floor and Akhtar 2006; Arunachalam 2013; Fitch et al. 2020; Foushee et al. 2021; Foushee and Srinivasan 2024). However, the impact of child‐surrounding speech on language development is still largely unexplored.

In addition, cultural differences further affect the availability of different types of input to the child and the number and type of speakers providing input. Ethnographic studies suggest that many cultures commonly share childcare responsibilities among several individuals, including neighbors, family members, or peers (Geertz 1961; Martini and Kirkpatrick 1992; Rogoff 1981; Lew‐Levy et al. 2020). In other, typically Western cultural settings, childcare is shared mainly between parents and childcare institutions.

Despite this large variability of sociocultural contexts in which children grow up, language acquisition research has mainly focused on child‐directed speech from adults, often centering specifically on maternal input. However, given the substantial cross‐cultural variation in input composition, concentrating only on (directed) input from adults might not be sufficient to understand how children in different cultural settings acquire language (Casillas 2023). In many cultures, children are exposed to large amounts of speech from other children, both directly addressed to them and in the surrounding environment. However, only a few studies have systematically explored this type of input (Loukatou et al. 2022; Cristia et al. 2023).

Several indicators suggest that child speech might function as a significant source of input for language learning in children. First, child speech shares several acoustic and structural characteristics with child‐directed speech (Lee et al. 1999; Potamianos and Narayanan 2007). Second, pre‐babbling infants prefer to listen to infant vowel sounds over adult vowel sounds (Masapollo et al. 2016). Third, the presence of siblings is positively associated with the acquisition of pronouns of second‐born children (Oshima‐Takane et al. 1996). Finally, the linguistic context of child speech provides more relevant topics, tends to reference the “here and now” more frequently, and relates more to children's immediate experiences than adult speech.

Here, we investigated the effect of speech of other children on infant attention in two studies. In Study 1, we asked whether infants are more attentive when listening to surrounding speech from other children than to surrounding speech from adults (not child‐directed). We compared infants growing up in two diverse cultures (Shipibo‐Konibo, Peruvian Amazon and Swiss German, Zurich). This allows us to understand better how diverse socioecologies of childhood Kline et al. (2018) affect the attention of infants to different types of speech. We predicted that surrounding speech of children captures the attention of infants more than surrounding speech of adults. There are substantial acoustic similarities between child speech and child‐directed speech, suggesting that they have a similar effect on children's attention. Therefore, in Study 2, we asked whether infants are more attentive when listening to speech from other children compared to child‐directed speech from adults. In Study 2, we tested only Swiss German infants.

### 1.1 Cultural Contexts of This Study

The selection of Shipibo‐Konibo (Peru) and Swiss German was driven by the observation that the two cultures show substantial differences regarding the linguistic and cultural environment to which infants are exposed in their daily lives. In addition, the likelihood that infants from one culture have encountered the language of the other culture is very low, which is a critical factor in the experimental design.

#### 1.1.1 Shipibo‐Konibo

Shipibo‐Konibo is a Panoan language spoken by approximately 30,000 people in the Peruvian Amazon in the Ucayali River valley (Valenzuela 2003). Most speakers are bilingual, with Spanish as their second language, but children usually learn Shipibo‐Konibo as their first language. After entering primary school, which is bilingual, most children start to speak Spanish. The community lives in approximately 130 separate villages along the Ucayali River valley. With a few exceptions, these villages can only be reached by boat. Related nuclear families largely form villages. One or sometimes more families live together in one house, spanning two or three generations (Valenzuela 2003). As in many other Amazonian cultures, child‐led alloparenting is very common (Mezzenzana 2020). From early on, children often play in large groups, with older children taking care of younger ones (De Carvalho Rodrigues Lopes 2022). Independence is encouraged from an early age. Young toddlers are promoted to follow their slightly older peers in their daily activities and move around freely without supervision by adults (De Carvalho Rodrigues Lopes 2022). In our sample, data collection took place in three villages, all connected through family bonds.

#### 1.1.2 Switzerland

Swiss German is a continuum of Alemannic dialects spoken in the German‐speaking part of Switzerland. It is part of the Indo‐European language family (Glaser 2003). Swiss German infants were recruited in the urban area of Zurich. They all acquired Swiss German as their dominant language. Zurich is a highly urban and industrialized area. Core families mostly live in apartments or individual houses. On average, there are 1.5 children per family in Zurich (Craviolini 2020). The primary caregivers are parents, but a large proportion of children also attend institutional and non‐institutional external childcare. According to an overview of the Swiss Federal Statistical Office from 2021, approximately 63.9% of 0–3 year‐olds in Switzerland attend external childcare at least once a week, with 34.3% of children attending institutional childcare and 36.1% being taken care of by grandparents (Bundesamt für Statistik 2022).

## 2 Study 1

### 2.1 Materials and Methods

#### 2.1.1 Participants

The first study involved 127 infants aged 8–20 months. We tested 67 infants growing up in the Shipibo‐Konibo community in Peru (37 girls, age range: 8–20 months; mean age: 13.3 months; average number of siblings: 3.85, range of siblings: 0 to 10). Twelve additional infants also participated in the experiment but were excluded from further analyses due to fussiness (*n* = 10), falling asleep (*n* = 1), or equipment failure (*n* = 1). Two research assistants from the community and the first author recruited infants in Peru in three different villages in the Ucayali River valley. All mothers spoke Shipibo‐Konibo as their primary language. Infants received a small gift for their participation. Caregivers additionally received a small financial compensation for their participation.

In addition, we tested 60 infants growing up in Switzerland (area of Zurich, 29 girls, age range: 8–20 months; mean age: 13.9 months; average number of siblings: 0.47, range of siblings: 0 to 2). Twelve additional infants also participated in the experiment but were excluded from further analyses due to fussiness (*n* = 12). Infants in Switzerland were recruited through the daycare center network of the University of Zurich. The study was conducted in six daycare centers. All infants received a small gift for their participation.

Data collection in both places took place from September 2022 until April 2023. Data in Peru was collected first. The age distributions of the Shipibo‐Konibo sample were matched with the Swiss sample as closely as possible. All protocols were approved by the ethics committee of the Faculty of Arts and Social Sciences of the University of Zurich (approval nr. 19.6.9 and 22.6.13). All parents gave their written consent to participate in the study. In Peru, permission to conduct this research was obtained from representatives from all three villages.

#### 2.1.2 Stimuli

Stimuli sets in both languages consisted of 12 conversation snippets extracted from naturalistic language corpora that the first author collected. Data for the stimuli recordings was collected using a Zoom Q8 video recorder placed on a tripod with a distance to the speakers of approximately four meters. Each stimulus has a duration of 25 s. For both languages, six stimuli consisted of child speech (children aged 3.5–5 years) and six stimuli consisted of adult speech (2*female‐female, 2*male‐male, 2*mixed; aged 25–35 years). We opted for purely naturalistic stimuli to create an ecologically valid setup, which mimics real‐world conversations as closely as possible, as opposed to artificial or seminatural dialogues or monologues. All stimuli were equated in mean amplitude. Additional details of the acoustic characteristics of the stimulus set can be found in Table 1. Measurements were made using the software Praat (Version 6.4.06) (Boersma 2001), using default values to extract the fundamental frequency (F0). More descriptive information about the stimuli set can be found in the supplementary materials (see Table S1).

**TABLE 1:** Stimuli characteristics of child and adult speech used in Study 1. F0 equals fundamental frequency.

	**Child speech**				**Adult speech**			
	Swiss German		Shipibo‐Konibo		Swiss German		Shipibo‐Konibo	
**Feature**	*M*	*SD*	*M*	*SD*	*M*	*SD*	*M*	*SD*
F0 mean (Hz)	209.75	87.34	237.06	84.73	194.0	17.9	175.9	52.0
F0 max (Hz)	465.60	76.14	504.32	74.60	405.2	71.2	420.9	81.0
F0 min (Hz)	84.00	17.79	79.00	20.15	67.80	11.10	53.00	5.17
F0 range (Hz)	381.6	78.19	425.32	77.27	337.4	72.06	367.9	81.16
Mean *n* of	9.5	1.4	10.0	1.4	7.5	1.4	7.0	0.9
utterances								

#### 2.1.3 Procedure

Infants sat on their caregiver's lap at a table. Two 13‐in. monitors (2560‐by‐1600 Apple retina display) were placed to the left and right of the infant at a 120‐degree angle approximately 60 cm away. A zoom Q8 video recorder was placed between the two monitors to record the infants' looking times for later manual coding. The experimenter was hidden behind a portable wall and controlled the presentation of the stimuli. Two familiarization trials (pinwheel with classical music on each screen) were presented before the critical trials began.

For each trial, the audio stimulus was presented through the monitors, while a spinning pinwheel was shown on the screen. A total of 12 stimuli were presented in random order. The stimulus and pinwheel presentation stopped whenever the infant looked away from the screen for more than two seconds. A blinking LED light centered between the two monitors appeared to direct the infant's attention back to the center. Participants were tested in a between‐subject design. In both cultures, half of the participants were presented with stimuli in their native language. In contrast, the other half were presented with stimuli in the respective unknown language (Swiss German or Shipibo‐Konibo). Caregivers were instructed to look straight at the camera and not interact with the child.

Several measurements were taken to ensure that the test environments in Peru were as comparable as possible to the setup in Switzerland. In all three villages in Peru, the experiment was set up inside a house (family home in two cases or a communal house in one case) with no other people but the first author present. The experimental setup was arranged on a table facing a wall without windows to minimize visual distractions. Identical equipment, including a portable wall, two screens, a distractor light, and a camera, was used in all locations. In all locations, the distance between the table and wall, the chair and table, and the distance between screens and distractor lights were carefully controlled. To further ensure consistency, the first author conducted the procedure in all cases, ensuring uniform instructions for caregivers. As the use of headphones was not culturally appropriate for Shipibo‐Konibo caregivers in the context of our study, we provided clear verbal instructions, asking them to maintain direct eye contact with the camera throughout the experiment and not interact with the child. To ensure a comparable setup, we also chose not to provide headphones to the Swiss caregivers.

#### 2.1.4 Manual Coding

The first author coded infants' looking times for each trial based on video recordings, using ELAN software ELAN (Version 6.7) (2023) (Version 6.7). A naïve second coder double‐coded 15% of all trials. Inter‐rater reliability was high (ICC = 0.91).

#### 2.1.5 Statistical Analysis

We fitted a Bayesian generalized mixed model to examine differences in looking times between participant groups and stimuli types through the brms Bürkner (2017) interface to Stan Carpenter et al. (2017) in *R* (Version 4.3.2) (R Core Team (Version 4.3.2) 2021). We included stimulus language (native/foreign), age (in months), culture, interacting with stimulus type (adult/child), interacting with number of siblings as fixed effects. Child ID was included as a random effect.

We choose weakly informative priors for the slope, Normal (μ = 0, σ = 1), and an Exponential (λ = 1) for the standard deviation of the random effects. Parameters were estimated by running four independent Monte Carlo Markov Chains for 4000 iterations each. We assessed the overall model performance by performing posterior predictive checks, and by calculating a Bayesian $R^{2}$‐statistic (Gelman et al. 2019). Chain convergence, mixture and stationarity were confirmed by visual inspection of trace plots and by ensuring that all $\hat{R}$= 1.00.

Bayesian methods estimate a posterior distribution that represents a range of plausible values for each estimated parameter. When reporting the parameter estimates of interest, we provide the posterior mean, the 95% highest density interval (HDI), and the probability of direction ($P(\hat{\beta }>0)$). We consider 95% (or greater) of the HDI (e.g., $P(\hat{\beta }>0)$ = 99.9%) in the predicted direction as strong evidence of an effect. We treat 89% (or greater) of the posterior distribution in the predicted direction as weak evidence of an effect, and less than 89% as no evidence. To quantify differences in looking time between adult and child stimuli, we calculate post‐hoc contrasts with the expected values of the posterior predictive distribution and report these in the main text (see Figure 3). The full outcome of all analyses can be found in the supplementary materials (see Figures S1 and S2). Data and scripts are available on the Open Science Framework repository: https://osf.io/dercj/?view_only=91db649c7a0b47f38be9067145833f0d.

**FIGURE 1:**
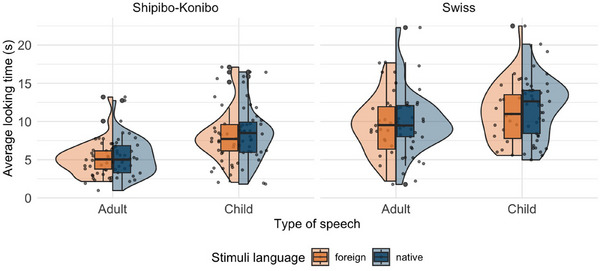
**Results of the headturn preference paradigm (Study 1)**. Infants' average looking times (in seconds) to surrounding child speech versus surrounding adult speech separated by culture and stimuli language.

**FIGURE 2:**
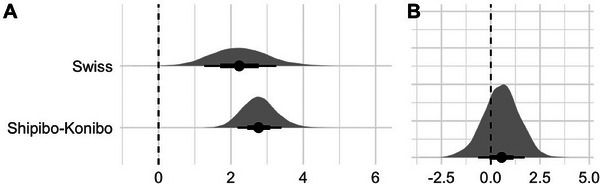
**Results of the generalized mixed model (Study 1)**. **(A**) Posterior distributions and densities of the expected difference in looking times between adult and child stimuli across cultures. **(B**) Posterior distributions and densities of the difference of looking time differences between the two cultures. Black horizontal bars indicate 50% and 80% credible intervals.

**FIGURE 3:**
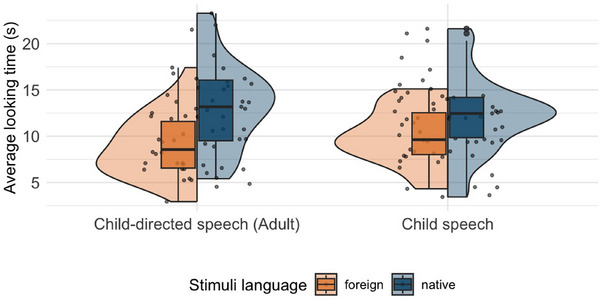
**Results of the headturn preference paradigm (Study 2)**. Infants' average looking times (in seconds) to child speech versus child‐directed speech from adults separated by stimuli language.

### 2.2 Results

Looking times from the head‐turn experiment of Study 1 are shown in Figure. 1. Across all conditions and participants, infants completed on average 10.5 trials. In the Shipibo‐Konibo sample, the mean looking time across all trials was 5.23 s for adult speech and 8.22 s for child speech. Mean looking time of the Swiss German sample was 9.77 s for adult speech and 11.51 s for child speech.

Looking at the Shipibo‐Konibo sample, results from our first model suggest that there is strong evidence for a difference in looking times toward child versus adult stimuli (*M* = 2.78, 95% HDI [1.89, 3.75], $P(\hat{\beta }>0)$ = 100%; see Figure 2A). This suggests that Shipibo‐Konibo infants paid more attention to surrounding child speech than to surrounding adult speech. Looking at the results from the Swiss sample, results again indicated a positive effect of stimulus type on infants' looking times (*M* = 2.25, 95% HDI [0.73, 3.83], $P(\hat{\beta }>0)$: 99%, see Figure 2A). This suggests that there is evidence for a difference in looking times in both cultures. When contrasting the looking time differences between child versus adult stimuli from both participants groups, results further indicated no evidence for cultural differences when comparing the effect size (*M* = 0.53, 95% HDI [‐1.26, 2.35], $P(\hat{\beta }>0)$ = 73%, see Figure 2B). This suggests that there are no cultural differences in the effect of stimuli type on looking time.

Results further indicated no evidence for an effect of stimulus language in both cultures (*Shipibo‐Konibo*: *M* = 0.16, 95% HDI [‐0.72, 1.00], $P(\hat{\beta }>0)$: 65%; *Swiss*: *M* = 0.28, 95% HDI [‐1.13, 1.70], $P(\hat{\beta }>0)$: 65%), which suggests that in both cultures infants showed similar looking times independent of the stimulus language they were exposed to. In addition, we found weak evidence for a positive age effect in both participant groups (Shipibo‐Konibo: *M* = 0.09, 95% HDI [‐0.04, 0.23], $P(\hat{\beta }>0)$: 92.2%; Swiss German: *M* = 0.14, 95% HDI [‐0.06, 0.34], $P(\hat{\beta }>0)$: 92.2%), suggesting that infants showed longer looking times with increasing age.

A total of four participants in the Shipibo‐Konibo sample grew up in the same village in which the Shipibo‐Konibo stimuli were recorded. We ran an additional analysis excluding data from these four participants to assess whether our findings remain robust when accounting for this factor. Results from the additional analysis suggest that our findings remain consistent when excluding these four participants (see S3 Supplementary Analysis).

### 2.3 Discussion

The results from Study 1 suggest that the surrounding speech from older children captures the attention of infants more than the surrounding speech from adults. By comparing infants from the Shipibo‐Konibo community in the Peruvian Amazon and infants growing up in an urban area in Switzerland, we demonstrate that the effect of child speech remained consistent across two distinct and unrelated cultures. Our results also indicate no cultural differences when comparing the effect size. This suggests that despite the different sociocultural environments, the two participant groups did not show differences in the attentional behaviors tested in the present study.

## 3 Study 2

Given the results from Study 1, we asked in Study 2 whether child‐directed speech captures infants' attention more effectively than child speech. In Study 2, we focused exclusively on Swiss German infants.

### 3.1 Materials and Methods

#### 3.1.1 Participants

A total of 61 infants aged 8–20 months participated in Study 2. We tested infants growing up in Switzerland (area of Zurich) (28 girls, age range: 8–20 months; mean age: 14.1 months; mean number of siblings: 0.44, range of siblings: 0 to 4; mean days at external daycare per week: 1.4). Fifteen additional infants participated in the experiment but were excluded from further analyses due to fussiness (*n* = 13) or equipment failure (*n* = 2). The infants were recruited through the participant database of the Research Unit Developmental Psychology: Infancy and Childhood at the University of Zurich, and the study was conducted in the Research Unit's facilities of the Department of Psychology. All infants received a small gift for their participation. Data collection took place between October 2023 and January 2024.

#### 3.1.2 Stimuli

The stimulus sets in both languages consisted of 12 speech snippets from 12 individual speakers extracted from the same corpora mentioned in Study 1. Each stimulus has a duration of 25 s. For both languages, six stimuli consisted of child speech (children aged 4–5 years), and six stimuli consisted of child‐directed adult speech (3*female, 3*male; aged 25–35 years). All stimuli were extracted from interactions between a single speaker (adult or child) and a 1‐year‐old child in a play context. All stimuli were equated in mean amplitude. Table 2 provides additional details of the stimulus set. Measurements were made with Praat (Version 6.4.06) (Boersma 2001), using default values to extract the fundamental frequency (F0). More descriptive information about the stimuli set can be found in the supplementary materials (see Table S2).

**TABLE 2:** Stimuli characteristics of child speech and child‐directed speech from adults used in Study 2. F0 equals fundamental frequency.

	**Child speech**				**Child‐directed speech**			
	Swiss German		Shipibo‐Konibo		Swiss German		Shipibo‐Konibo	
**Feature**	*M*	*SD*	*M*	*SD*	*M*	SD	*M*	*SD*
F0 mean (Hz)	241.60	74.09	251.82	81.01	222.9	51.5	203.5	48.7
F0 max (Hz)	513.76	95.3	507.65	90.7	413.0	22.6	417.8	30.5
F0 min (Hz)	88.51	20.21	101.29	41.67	76.1	36.2	81.0	21.4
F0 range (Hz)	425.25	97.42	406.36	99.81	336.9	42.68	336.8	37.26
Mean *n* of	9.7	1.2	10.0	1.7	10.0	1.9	10.0	1.9
utterances								

#### 3.1.3 Procedure

Participants were tested in a between‐subjects design. We used the same setup, and procedure as presented in Study 1.

#### 3.1.4 Manual Coding

Infants' looking times for each trial were coded by the first author based on video recordings, using ELAN software ELAN (Version 6.7) (2023) (Version 6.7). A naïve second coder double‐coded 15 % of all trials. Inter‐rater reliability was high (ICC = 0.93).

#### 3.1.5 Statistical Analysis

We fitted a second Bayesian mixed‐effects model through the brms Bürkner (2017) interface to Stan Carpenter et al. (2017) in R Core Team (Version 4.3.2) (2021) (Version 4.3.2) to analyze infants' looking times. We included stimulus language (foreign/native), age (in months), stimulus type (child/adult), number of siblings and number of days spent at daycare as fixed effects. Child ID was included as a random effect. Parameters were estimated by running four independent Monte Carlo Markov Chains for 4000 iterations each. We choose weakly informative priors for the slope, Normal (μ = 0, σ = 1), and an Exponential (λ = 1) for the standard deviation of the random effects. We assessed the overall model performance by performing posterior predictive checks, and by calculating a Bayesian $R^{2}$‐statistic (Gelman et al. 2019). Chain convergence, mixture and stationarity were confirmed by visual inspection of trace plots, and by ensuring that all $\hat{R}$= 1.00. Similar to the first model, contrasts were calculated with the expected values of the posterior predictive distribution.

### 3.2 Results

Looking times from the head‐turn experiment of Study 2 are shown in Figure 3. Across both conditions, infants completed on average 9.6 trials. The mean looking time across all infants and trials was 11.16 s for adult speech and 11.27 s for child speech.

**FIGURE 4:**
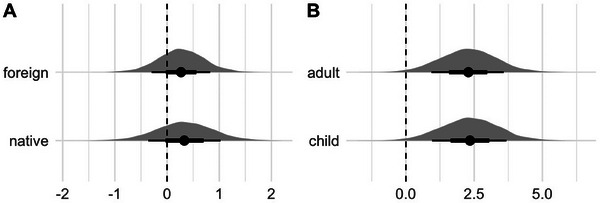
**Results of the generalized mixed model (Study 2)**. **(A)** Posterior distributions and densities of the expected difference in looking times between adult and child stimuli across both conditions (foreign vs. native language stimuli). **(B)** Posterior distributions and densities of the expected difference in looking times across condition (foreign vs. native language stimuli) for both stimuli types (adult/child). Black horizontal bars indicate 50% and 80% credible intervals.

The analysis showed that in both conditions, we do not find evidence for a difference between looking times toward child‐directed speech and child speech (*Foreign*: *M* = 0.27, 95% HDI [‐0.58, 1.19], $P(\hat{\beta }>0)$: 73%; *Native*: *M* = 0.33, 95% HDI [‐0.76, 1.43], $P(\hat{\beta }>0)$: 73%, see Figure 4A). Results further revealed that the effect of stimulus language had a high probability of being positive (*M* = 2.31, 95% HDI [0.20, 4.37], $P(\hat{\beta }>0)$: 98.4%, see Figure 4). This suggests that overall, infants paid more attention to stimuli in Swiss German compared to stimuli in Shipibo‐Konibo. Furthermore, we found no evidence for an age effect (*M* = ‐0.18, 95% HDI [‐0.5 0.13], $P(\hat{\beta }>0)$: 12.3%).

## 4 General Discussion

In Study 1, we compared infants from the Shipibo‐Konibo community in the Peruvian Amazon with infants growing up in an urban area in Switzerland. The results show that surrounding speech from other children (not addressed to the child) captivates infants' attention more than surrounding speech from adults (not addressed to the child) across cultures. The effect of child speech remains consistent despite the diverse sociolinguistic environments of our two participant groups.

In Study 2, we examined whether child speech has a similar effect as the well‐known and cross‐culturally established effect of child‐directed speech from adults (e.g., *ManyBabies* 2020). We focused on Swiss German, where infants typically are less exposed to other children. Our results show that child speech captured the attention of Swiss German infants at the same level as child‐directed speech from adults.

Some previous studies on the role of older children's speech in language acquisition suggest that input from older siblings does not contribute positively to language development as it is has been argued to be less useful in the pragmatic domain (Tomasello and Mannle 1985), and can potentially result in fewer resources being allocated to each child (Peyre et al. 2016). In contrast, other studies highlight the benefits of older siblings' speech in specific areas, such as pronoun acquisition Oshima‐Takane et al. (1996) and the development of sociocommunicative skills (Dunn and Kendrick 1982; Hoff 2006). Our results provide further evidence that, if available, (directed) input from older children may have the potential to contribute positively to language acquisition. This is further supported by the finding that child speech and child‐directed speech share several acoustic and structural characteristics, such as higher pitch or short utterances (Lee et al. 1999; Potamianos and Narayanan 2007), and the strong association between language development and child‐directed speech (e.g., Huttenlocher et al. 2010; Rowe 2012; Weisleder and Fernald 2013; Newman et al. 2016).

However, it remains to be tested whether the effect of Study 2 is similar in infants from a society with a lower prevalence of child‐directed speech from adults compared to the Swiss sample tested in this study. Previous research has shown the attention‐capturing effect of child‐directed speech in various cultures (Werker et al. 1994; Hayashi et al. 2001; ManyBabies 2020). More research on cultures showing smaller amounts of child‐directed speech from adults is still needed to test whether we find a similar pattern across non‐industrialized, more traditional cultures, where children spend a substantial amount of time with other children.

What drives the attention to child speech remains an open question. With respect to the acoustic features of our stimuli set (see Table 1 and Table 2), child speech in both languages shows higher F0 means compared to adult speech and slightly higher F0 means compared to child‐directed speech from adults in both languages. Taking the results from both studies together, however, we suggest that higher pitch is not the only factor that leads to increased attention to child speech. Presumably, there is also a social component that interacts with the higher pitch, providing an additional explanation for our findings: There is a growing literature demonstrating the social function of peers and older children in child development (Bailey et al. 1993; Rubin et al. 2011; Zmyj et al. 2012; Zmyj and Seehagen 2013). The observed phenomena related to children's inclination to imitate, observe, and learn from their peers and older children seems to be reflected in the findings of both of our studies. This is further supported by the finding that stimulus language did not affect infants' attention in Study 1, in which participants were exposed to surrounding child versus adult speech. This suggests that in this context, the linguistic content did not play a significant role in capturing their attention, but rather, other language‐independent acoustic characteristics of child speech.

The observed language effect in Study 2, characterized by higher attention to stimuli in the participants' native language, could be attributed to the stimuli. In this setting, participants were exposed to speech streams from single speakers. Here, children presumably felt directly addressed rather than observing an interaction as in Study 1. This might be a possible explanation for the increased attention toward stimuli presented in their native language. This further aligns with previous research that showed that infants in their first and second year prefer child‐directed speech in their native language compared to a foreign language (ManyBabies 2020).

To conclude, our results provide evidence that child speech has an enhanced impact on the attention of language‐learning children across distinct cultures. Whether this more substantial attention leads to better learning remains to be tested in future research. However, attention is at least a precondition for learning (Vouloumanos and Curtin 2014; Foushee et al. 2021). Our results, therefore, suggest that child speech might have an important impact on language development. In light of previous research on the role of child speech in language acquisition (Tomasello and Mannle 1985; Oshima‐Takane et al. 1996; Shneidman et al. 2013), our findings underscore the need to further explore which specific aspects of language acquisition may benefit from input from other children and at what stages of development this occurs.

The role of other children in the developmental process could potentially also explain why we do not see many cross‐linguistic or cross‐cultural differences in developmental processes of language learning even if the linguistic environments are highly diverse.

More cross‐cultural research that extends beyond urban and Western populations is needed to gain a more comprehensive understanding of language acquisition and the impact of different linguistic environments on the acquisition process.

## Author Contributions

**Johanna Schick**: conceptualization, data collection, data preparation, data analysis, writing – original draft, review and editing. **Moritz M. Daum**: resources, writing – review and editing. **Sabine Stoll**: conceptualization, funding acquisition, writing – review and editing.

## Conflicts of Interest

The authors declare no competing financial interests.

## Supporting information

desc70045‐sup‐0001‐SuppMat.pdf

desc70045‐sup‐0002‐SuppMat.zip

## Data Availability

The data and analyses scripts that support the findings of this study are available on the OSF repository: https://osf.io/dercj/. The audio stimuli used in this study cannot be publicly shared due to privacy and ethical concerns. Access to the stimuli may be granted under specific conditions upon request to the first author.

## References

[desc70045-bib-0001] Arunachalam, S. 2013. “Two‐Year‐Olds Can Begin to Acquire Verb Meanings in Socially Impoverished Contexts.” Cognition 129: 569–573. 24055833 10.1016/j.cognition.2013.08.021

[desc70045-bib-0002] Bailey, D. B. , M. R. Burchinal , and R. A. McWilliam . 1993. “Age of Peers and Early Childhood Development.” Child Development 64: 848–862. 8339699 10.1111/j.1467-8624.1993.tb02947.x

[desc70045-bib-0003] Casillas Bergelson, E. M. , M. Soderstrom , A. Seidl , A. S. Warlaumont , and A. Amatuni . 2019. “What Do North American Babies Hear? A Large‐Scale Cross‐Corpus Analysis.” Developmental Science 22: e12724. 30369005 10.1111/desc.12724 PMC6294666

[desc70045-bib-0004] Boersma, P. 2001. “Praat, A System for Doing Phonetics by Computer.” Glot International 5: 341–345.

[desc70045-bib-0005] Bundesamt für Statistik . 2022. “Anteil der familienergänzend betreuten Kinder im Alter von 0‐12 Jahren nach verschiedenen soziodemografischen Merkmalen.” https://bfs.admin.ch/asset/de/31786778.

[desc70045-bib-0006] Bürkner, P.‐C. 2017. “brms: An R Package For Bayesian Multilevel Models Using Stan.” Journal of Statistical Software 80: 1–28.

[desc70045-bib-0007] Carpenter, B. , A. Gelman , M. D. Hoffman , et al. 2017. “Stan: A Probabilistic Programming Language.” Journal of Statistical Software 76: 1–32. 36568334 10.18637/jss.v076.i01 PMC9788645

[desc70045-bib-0008] Casillas, M. 2023. “Learning Language in Vivo.” Child Development Perspectives 17: 10–17.

[desc70045-bib-0009] Casillas, M. , P. Brown , and S. C. Levinson . 2020. “Early Language Experience in a Papuan Community.” Journal of Child Language 48: 792–814. 32988426 10.1017/S0305000920000549

[desc70045-bib-0010] Cooper, R. P. , and R. N. Aslin . 1990. “Preference for Infant‐Directed Speech in the First Month After Birth.” Child Development 61: 1584–1595. 2245748

[desc70045-bib-0011] Correa‐Chávez, M. , and B. Rogoff . 2009. “Children's Attention to Interactions Directed to Others: Guatemalan Mayan and European American Patterns.” Developmental Psychology 45: 630–641. 19413421 10.1037/a0014144

[desc70045-bib-0012] Craviolini, J. 2020. “Viele Kinder Auf Dem Land – Wenige In Der Stadt. Eine Analyse Der räumlichen Unterschiede Des Geburtsverhaltens Im Kanton Zürich.” *Statistisches Amt Kanton Zürich*: 1–15.

[desc70045-bib-0013] Cristia, A. 2023. “A Systematic Review Suggests Marked Differences in the Prevalence of Infant‐Directed Vocalization Across Groups of Populations.” Developmental Science 26: e13265. 35429106 10.1111/desc.13265

[desc70045-bib-0014] Cristia, A. , L. Gautheron , and H. Colleran . 2023. “Vocal Input and Output Among Infants in a Multilingual Context: Evidence From Long‐Form Recordings in Vanuatu.” Developmental Science 26: e13375. 36751861 10.1111/desc.13375

[desc70045-bib-0015] Cristia, A. , M. Gurven , and J. Stieglitz . 2019. “Child‐Directed Speech Is Infrequent in a Forager‐Farmer Population: A Time Allocation Study.” Journal of Child Development 90: 759–773. 29094348 10.1111/cdev.12974 PMC8030240

[desc70045-bib-0055] De Carvalho Rodrigues Lopes, T . 2022. Childhood and Neo‐Extractive Development: Shipibo Children's Shifting Livelihoods and Social Protection in the Peruvian Amazonia. University of East Anglia.

[desc70045-bib-0016] Dunn, J. , and C. Kendrick . 1982. “The Speech of Two‐And‐Three‐Year‐Olds to Infant Siblings: ‘Baby Talk’ and the Context of Communication.” Journal of Child Language 9: 579–595. 7174758 10.1017/s030500090000492x

[desc70045-bib-0017] ELAN (Version 6.7) . 2023. Nijmegen: Max Planck Institute for Psycholinguistics. The Language Archive.

[desc70045-bib-0018] Fernald, A. 1985. “Four‐Month‐Old Infants Prefer to Listen to Motherese.” Infant Behavior and Development 8: 181–195.

[desc70045-bib-0019] Fitch, A. , A. M. Lieberman , R. J. Luyster , and S. Arunachalam . 2020. “Toddlers' Word Learning Through Overhearing: Others' Attention Matters.” Journal of Experimental Child Psychology 193: 104793. 31992441 10.1016/j.jecp.2019.104793 PMC7114829

[desc70045-bib-0020] Floor, P. , and N. Akhtar . 2006. “Can 18‐Month‐Old Infants Learn Words by Listening in on Conversations?.” Infancy 9: 327–339. 33412677 10.1207/s15327078in0903_4

[desc70045-bib-0021] Foushee, R. , and M. Srinivasan . 2024. “Infants Who Are Rarely Spoken to Nevertheless Understand Many Words.” Proceedings of the National Academy of Sciences 121: e2311425121. 10.1073/pnas.2311425121 PMC11161804 38814865

[desc70045-bib-0022] Foushee, R. , M. Srinivasan , and F. Xu . 2021. “Self‐Directed Learning by Preschoolers in a Naturalistic Overhearing Context.” Cognition 206: 104415. 33075567 10.1016/j.cognition.2020.104415

[desc70045-bib-0023] Gaskins, S. 2020. “Cultural Perspectives on Infant Caregiver Interaction.” Roots of Human Sociality 279–298.

[desc70045-bib-0024] Geertz, H. 1961. “The Javanese Family: A Study of Kinship and Socialization.” Waveland Press.

[desc70045-bib-0025] Gelman, A. , B. Goodrich , J. Gabry , and A. Vehtari . 2019. “R‐squared for Bayesian Regression Models.” American Statistician 73: 307–309.

[desc70045-bib-0026] Glaser, E. 2003. Gömmer MiGro, edited by B. Dittli , A. Häcki Buhofer , and W. Haas , 39–66. Universitätverlag.

[desc70045-bib-0027] Hayashi, A. , Y. Tamekawa , and S. Kiritani . 2001. “Developmental Change in Auditory Preferences for Speech Stimuli in Japanese Infants.” Journal of Speech, Language, and Hearing Research 44: 1189–1200. 10.1044/1092-4388(2001/092) 11776357

[desc70045-bib-0028] Hoff, E. 2006. “How Social Contexts Support and Shape Language Development.” Developmental Review 26: 55–88.

[desc70045-bib-0029] Huttenlocher, J. , H. Waterfall , M. Vasilyeva , J. Vevea , and L. V. Hedges . 2010. “Sources of Variability in Children's Language Growth.” Cognitive Psychology 61: 343–365. 20832781 10.1016/j.cogpsych.2010.08.002 PMC2981670

[desc70045-bib-0030] Keller, H. 2022. Cultures of Infancy. Routledge.

[desc70045-bib-0031] Kidd, E. , and R. Garcia . 2022. “How Diverse Is Child Language Acquisition Research?.” First Language 42: 703–735. 10.1177/01427237221100138 PMC9605524 36310838

[desc70045-bib-0032] Kline, M. A. , R. Shamsudheen , and T. Broesch . 2018. “Variation is the Universal: Making Cultural Evolution Work in Developmental Psychology.” Philosophical Transactions of the Royal Society B: Biological Sciences 373: 20170059. 10.1098/rstb.2017.0059 PMC5812971 29440524

[desc70045-bib-0033] Lee, S. , A. Potamianos , and S. Narayanan . 1999. “Acoustics of Children's Speech: Developmental Changes of Temporal and Spectral Parameters.” Journal of the Acoustical Society of America 105: 1455–1468. 10089598 10.1121/1.426686

[desc70045-bib-0034] Lew‐Levy, S. , S. M. Kissler , A. H. Boyette , A. N. Crittenden , I. A. Mabulla , and B. S. Hewlett . 2020. “Who Teaches Children to Forage? Exploring the Primacy of Child‐To‐Child Teaching Among Hadza and Bayaka Hunter‐Gatherers of Tanzania and Congo.” Evolution and Human Behavior 41: 12–22.

[desc70045-bib-0035] Loukatou, G. , C. Scaff , K. Demuth , A. Cristia , and N. Havron . 2022. “Child‐Directed and Overheard Input from Different Speakers in Two Distinct Cultures.” Journal of Child Language 49: 1173–1192. 34663486 10.1017/S0305000921000623

[desc70045-bib-0036] ManyBabies,. 2020. “Quantifying Sources of Variability in Infancy Research Using the Infant‐Directed‐Speech Preference.” Advances in Methods and Practices in Psychological Science 3: 24–52.

[desc70045-bib-0037] Martini, M. I. , and J. Kirkpatrick . 1992. “Parenting in Polynesia: A View From the Marquesas.” Annual Advances in Applied Developmental Psychology 5: 199–222.

[desc70045-bib-0038] Masapollo, M. , L. Polka , and L. Ménard . 2016. “When Infants Talk, Infants Listen: Pre‐Babbling Infants Prefer Listening to Speech With Infant Vocal Properties.” Developmental Science 19: 318–328. 25754812 10.1111/desc.12298

[desc70045-bib-0039] Mezzenzana, F. 2020. “Between Will and Thought: Individualism and Social Responsiveness in Amazonian Child Rearing.” American Anthropologist 122: 540–553.

[desc70045-bib-0040] Newman, R. S. , and I. Hussain . 2006. “Changes in Preference For Infant‐Directed Speech in Low and Moderate Noise By 4.5‐to 13‐Month‐Olds.” Infancy 10: 61–76. 33412673 10.1207/s15327078in1001_4

[desc70045-bib-0041] Newman, R. S. , M. L. Rowe , and N. Bernstein Ratner . 2016. “Input and Uptake at 7 Months Predicts Toddler Vocabulary: The Role of Child‐Directed Speech and Infant Processing Skills in Language Development.” Journal of Child Language 43: 1158–1173. 26300377 10.1017/S0305000915000446

[desc70045-bib-0042] Ochs, E. , and B. Schieffelin . 1984. Language acquisition and socialization: Three developmental stories and their implications. In:Culture Theory: Essays on Mind, Self and Emotion, edited by R. A. Shweder and R. A. LeVine , 276–320. Cambridge University Press.

[desc70045-bib-0043] Orena, A. J. , K. Byers‐Heinlein , and L. Polka . 2020. “What Do Bilingual Infants Actually Hear? Evaluating Measures of Language Input to Bilingual‐Learning 10‐Month‐Olds.” Developmental Science 23: e12901. 31505096 10.1111/desc.12901

[desc70045-bib-0044] Oshima‐Takane, Y. , E. Goodz , and J. L. Derevensky . 1996. “Birth Order Effects on Early Language Development: Do Secondborn Children Learn From Overheard Speech?.” Child Development 67: 621–634.

[desc70045-bib-0045] Peyre, H. , J. Y. Bernard , N. Hoertel , et al . 2016. “Differential Effects of Factors Influencing Cognitive Development at the Age of 5‐to‐6 Years.” Cognitive Development 40: 152–162.

[desc70045-bib-0046] Potamianos, A. , and S. Narayanan . 2007. A Review of the Acoustic and Linguistic Properties of Children's Speech. In *IEEE 9th Workshop on Multimedia Signal Processing* , 22–25.

[desc70045-bib-0047] R Core Team (Version 4.3.2) . 2021. R: A Language and Environment for Statistical Computing, R Foundation for Statistical Computing, Vienna, Austria.

[desc70045-bib-0048] Rogoff, B. 1981. “Adults and Peers as Agents of Socialization: A Highland Guatemalan Profile.” Ethos 9: 18–36.

[desc70045-bib-0049] Rowe, M. 2012. “A Longitudinal Investigation of the Role of Quantity and Quality of Child‐Directed Speech in Vocabulary Development.” Child Development 83: 1762–1774. 22716950 10.1111/j.1467-8624.2012.01805.x PMC3440540

[desc70045-bib-0050] Rubin, K. H. , W. M. Bukowski , and B. Laursen . 2011. Handbook of Peer Interactions, Relationships, and Groups. Guilford Press.

[desc70045-bib-0051] Shneidman, L. , and S. Goldin‐Meadow . 2012. “Language Input and Acquisition in a Mayan Village: How Important is Directed Speech?.” Developmental Science 15: 659–673. 22925514 10.1111/j.1467-7687.2012.01168.x PMC3538130

[desc70045-bib-0052] Shneidman, L. , J. S. Buresh , P. M. Shimpi , J. Knight‐Schwarz , and A. L. Woodward . 2009. “Social Experience, Social Attention and Word Learning in an Overhearing Paradigm.” Language Learning and Development 5: 266–281.

[desc70045-bib-0053] Shneidman, L. A. , M. E. Arroyo , S. C. Levine , and S. Goldin‐Meadow . 2013. “What Counts as Effective Input for Word Learning?.” Journal of Child Language 40: 672–686. 22575125 10.1017/S0305000912000141 PMC3445663

[desc70045-bib-0054] Sperry, D. E. , L. L. Sperry , and P. J. Miller . 2019. “Reexamining the Verbal Environments of Children From Different Socioeconomic Backgrounds.” Child Development 90: 1303–1318. 29707767 10.1111/cdev.13072

[desc70045-bib-0056] Tomasello, M. , and S. Mannle . 1985. “Pragmatics of Sibling Speech to One‐Year‐Olds.” Child Development 56, no. 4: 911–917.

[desc70045-bib-0057] Valenzuela, P. 2003. Transitivity in Shipibo‐Konibo Grammar: A Typologically Oriented Study. University of Oregon.

[desc70045-bib-0058] Vouloumanos, A. , and S. Curtin . 2014. “Foundational Tuning: How Infants' Attention to Speech Predicts Language Development.” Cognitive Science 38: 1675–1686. 25098703 10.1111/cogs.12128

[desc70045-bib-0059] Weisleder, A. , and A. Fernald . 2013. “Talking to Children Matters: Early Language Experience Strengthens Processing and Builds Vocabulary.” Psychological Science 24: 2143–2152. 24022649 10.1177/0956797613488145 PMC5510534

[desc70045-bib-0060] Werker, J. F. , J. E. Pegg , and P. J. McLeod . 1994. “A Cross‐Language Investigation of Infant Preference for Infant‐Directed Communication.” Infant Behavior and Development 17: 323–333.

[desc70045-bib-0061] Zmyj, N. , and S. Seehagen . 2013. “The Role of a Model's Age for Young Children's Imitation: A Research Review.” Infant and Child Development 22: 622–641.

[desc70045-bib-0062] Zmyj, N. , G. Aschersleben , W. Prinz , and M. Daum . 2012. “The Peer Model Advantage in Infants' Imitation of Familiar Gestures Performed by Differently Aged Models.” Frontiers in Psychology 3: 252. 22833732 10.3389/fpsyg.2012.00252 PMC3400440

