# Supplementary material for: Input to the Language Learning Infant: The Impact of Other Children
Source: Dev Sci. 2025 Jul 20;28(5):e70045. doi: 10.1111/desc.70045 (PMC12277873; doi:10.1111/desc.70045)
Supplement: Supplementary file 1 — desc70045‐sup‐0001‐SuppMat.pdf [file DESC-28-e70045-s001.pdf]

Supplementary Information for: ”Input to the language learning infant: The impact of other children”

Johanna Schick
Institute for the Interdisciplinary Study of Language Evolution,
University of Zurich
johanna.schick@uzh.ch

Moritz M. Daum
Department of Psychology,
University of Zurich
daum@psychologie.uzh.ch

Sabine Stoll
Institute for the Interdisciplinary Study of Language Evolution,
University of Zurich
sabine.stoll@uzh.ch

June 17, 2025

S1: Stimuli description

Study 1

Language	Stimuli number	Speaker gender	Age	Context	Number of utterances	Number of questions	Number of declaratives	Number of imperatives
SK	1	m+m	28, 30	traveling	7	0	7	0
SK	2	m+m	25, 31	traveling	7	3	3	1
SK	3	f+m	30, 35	traveling	6	0	5	1
SK	4	f+m	27, 31	traveling	8	1	7	0
SK	5	f+f	30, 31	traveling	8	1	7	0
SK	6	f+f	32, 33	traveling	6	0	5	1
SG	7	f+f	34, 35	traveling	7	2	5	0
SG	8	f+f	30, 35	traveling	6	2	4	0
SG	9	m+m	28, 31	traveling	9	1	8	0
SG	10	m+m	30, 30	traveling	8	1	6	1
SG	11	f+m	27, 33	traveling	9	1	7	1
SG	12	f+m	27, 29	traveling	6	0	6	0

Overview of stimuli set of surrounding adult speech for study 1. F=female, M=male. SK=Shipibo-Konibo, SG=Swiss-German

Language	Stimuli number	Speaker sex	Age	Context	Number of utterances	Number of questions	Number of declaratives	Number of imperatives
SK	1	m+m	3;7, 4;2	playing with car	9	4	2	3
SK	2	m+m	4;1, 4;2	playing in the kitchen	10	0	9	1
SK	3	f+m	3;10, 4;6	playing family	10	0	6	4
SK	4	f+m	4;6, 5;0	playing with shoes	8	0	6	2
SK	5	f+f	4;3, 4;11	playing with mirror	9	1	5	3
SK	6	f+f	3;10, 4;0	playing cooking	9	1	7	1
SG	7	f+f	4;10, 5;0	playing family	11	0	8	3
SG	8	f+f	4;6, 5;0	playing in sand	8	0	7	1
SG	9	m+m	3;11, 4;6	playing in sand	11	2	7	2
SG	10	m+m	3;7, 4;11	playing with water	9	1	7	1
SG	11	f+m	4;1, 4;10	playing in sand	8	0	7	1
SG	12	f+m	4;11, 5;0	playing in sand	10	3	6	1

Overview of stimuli set of surrounding child speech for study 1. F=female, M=male. SK=Shipibo-Konibo, SG=Swiss-German

Study 2

Language	Stimuli number	Speaker gender	Age (years)	Context	Number of utterances	Number of questions	Number of declaratives	Number of imperatives
SK	1	m	29	playing in kitchen	10	1	3	6
SK	2	m	28	playing with a ball	10	0	4	6
SK	3	m	35	playing with a bowl	9	0	4	5
SK	4	f	28	playing with clothes	10	1	6	3
SK	5	f	30	playing with a car	10	1	5	4
SK	6	f	33	playing in kitchen	10	2	6	4
SG	7	m	31	playing with a car	11	2	7	2
SG	8	m	35	unpacking a gift	8	1	6	1
SG	9	m	34	playing kitchen	8	0	6	2
SG	10	f	28	building a play house	10	4	5	1
SG	11	f	31	playing with a pin	13	5	6	2
SG	12	f	33	playing board game	11	3	5	3

Overview of stimuli set of child-directed speech from adults (single speakers) for study 2. F=female, M=male. SK=Shipibo-Konibo, SG=Swiss-German

Language	Stimuli number	Speaker sex	Age	Context	Number of utterances	Number of questions	Number of declaratives	Number of imperatives
SK	1	f	4;2	playing with a candle	10	0	7	3
SK	2	f	4;5	playing family	9	0	7	2
SK	3	f	4;11	playing with animals	10	0	7	3
SK	4	m	4;1	playing with clothes	11	1	7	3
SK	5	m	4;7	playing with ball	8	0	7	1
SK	6	m	4;6	playing with cups	10	0	6	4
SG	7	f	4;6	building st. out of sand	10	3	7	1
SG	8	f	4;11	playing family	9	0	6	3
SG	9	f	5;0	building st. out of sand	8	0	5	3
SG	10	m	4;6	playing shopping	9	0	7	2
SG	11	m	4;7	playing outside	11	1	6	4
SG	12	m	4;4	playing outside	11	0	8	3

Overview of stimuli set of child speech (single speakers) for study 2. F=female, M=male. SK=Shipibo-Konibo, SG=Swiss-German

S2: Model summaries

Model 1

```
##  Family: lognormal 
##   Links: mu = identity; sigma = identity 
## Formula: average ~ culture * stim + siblings + stim_language + months + (1 | ID) 
##    Data: df_first (Number of observations: 254) 
##   Draws: 4 chains, each with iter = 4000; warmup = 2000; thin = 1;
##          total post-warmup draws = 8000
## 
## Multilevel Hyperparameters:
## ~ID (Number of levels: 128) 
##               Estimate Est.Error l-95% CI u-95% CI Rhat Bulk_ESS Tail_ESS
## sd(Intercept)     0.25      0.05     0.15     0.33 1.00     1530     1412
## 
## Regression Coefficients:
##                        Estimate Est.Error l-95% CI u-95% CI Rhat Bulk_ESS
## Intercept                  1.27      0.18     0.92     1.63 1.00     5472
## cultureSwiss               0.68      0.11     0.46     0.90 1.00     4514
## stimchild                  0.44      0.07     0.31     0.58 1.00     7259
## siblings                   0.02      0.02    -0.03     0.06 1.00     4677
## stim_languagenative        0.03      0.07    -0.10     0.15 1.00     6288
## months                     0.01      0.01    -0.01     0.03 1.00     5971
## cultureSwiss:stimchild    -0.24      0.10    -0.42    -0.04 1.00     7458
##                        Tail_ESS
## Intercept                  5651
## cultureSwiss               4954
## stimchild                  5928
## siblings                   5393
## stim_languagenative        5957
## months                     6332
## cultureSwiss:stimchild     5808
## 
## Further Distributional Parameters:
##       Estimate Est.Error l-95% CI u-95% CI Rhat Bulk_ESS Tail_ESS
## sigma     0.39      0.03     0.34     0.44 1.00     2247     2392
## 
## Draws were sampled using sampling(NUTS). For each parameter, Bulk_ESS
## and Tail_ESS are effective sample size measures, and Rhat is the potential
## scale reduction factor on split chains (at convergence, Rhat = 1).
```

Figure S1: Model summary of the multilevel Bayesian model analyzing infant looking times toward child-surrounding speech from children vs. adults. CI = credible interval.

Model 2

```
##  Family: lognormal
##   Links: mu = identity; sigma = identity
## Formula: average ~ stim + siblings + daycare_days_per_week + months + stim_language + (1 | ID)
##    Data: df_second (Number of observations: 122)
##   Draws: 4 chains, each with iter = 4000; warmup = 2000; thin = 1;
##          total post-warmup draws = 8000
##
## Multilevel Hyperparameters:
## ~ID (Number of levels: 61)
##               Estimate Est.Error l-95% CI u-95% CI Rhat Bulk_ESS Tail_ESS
## sd(Intercept)     0.30      0.04     0.22     0.38 1.00     2610     4595
##
## Regression Coefficients:
##                       Estimate Est.Error l-95% CI u-95% CI Rhat Bulk_ESS
## Intercept                 2.43      0.23     1.98     2.89 1.00     3951
## stimchild                 0.03      0.05    -0.06     0.12 1.00    16602
## siblings                 -0.14      0.06    -0.26    -0.02 1.00     5053
## daycare_days_per_week     0.06      0.04    -0.02     0.14 1.00     4054
## months                   -0.02      0.01    -0.05     0.01 1.00     3926
## stim_languagenative       0.22      0.10     0.02     0.41 1.00     3957
##                       Tail_ESS
## Intercept                 5077
## stimchild                 4802
## siblings                  5762
## daycare_days_per_week     5206
## months                    5348
## stim_languagenative       5069
##
## Further Distributional Parameters:
##       Estimate Est.Error l-95% CI u-95% CI Rhat Bulk_ESS Tail_ESS
## sigma     0.26      0.02     0.22     0.31 1.00     3300     5366
##
## Draws were sampled using sampling(NUTS). For each parameter, Bulk_ESS
## and Tail_ESS are effective sample size measures, and Rhat is the potential
## scale reduction factor on split chains (at convergence, Rhat = 1).
```

Figure S2: Model summary of the multilevel Bayesian model analyzing infant looking times toward child-directed speech from adults vs. child speech. CI = credible interval.

S3: Supplementary analysis

The speech corpus used to extract the Shipibo-Konibo stimuli was collected in one of the three villages in which participants for Study 1 were recruited. As a result, participants from this village (n=4) were more likely to hear speech from potentially familiar speakers. To assess whether our findings remain robust when accounting for this factor, we conducted an additional analysis excluding the looking time data from these four partici-

pants.

Results from the additional analysis demonstrate that results from study 1 remain consistent when excluding these four participants. We found strong evidence for a difference in looking times towards child vs. adult stimuli for Shipibo-Konibo participants (mean = 3.20, 95% HDI [2.03, 4.38], $P(\hat{\beta} > 0)$ = 100%) and and weak evidence for a difference in looking times in Swiss participants (mean = 2.71, 95% HDI [-1.19, 7.10], $P(\hat{\beta} > 0)$ = 91%) (see Figure S3A). When contrasting the looking time differences between child vs. adult stimuli from both participants groups, results again indicate no evidence for cultural differences when comparing the effect size (mean = 0.48, 95% HDI [-4.12, 4.46], $P(\hat{\beta} > 0)$ = 60%) (see Figure S3B). The full model outcome can be found in Figure S4.

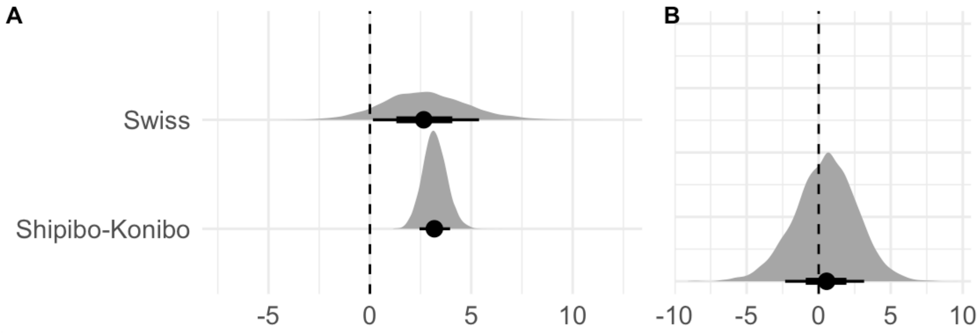

Figure S3: **A**: Posterior distributions and densities of the expected difference in looking times between adult and child stimuli across cultures. **B**: Posterior distributions and densities of the difference of looking time differences between the two cultures. Horizontal bars indicate 50% and 80% credible intervals.

```
##  Family: lognormal
##   Links: mu = identity; sigma = identity
## Formula: average ~ culture * (stim * siblings) + stim_language + months + (1 | ID)
##    Data: df_first (Number of observations: 246)
##   Draws: 4 chains, each with iter = 4000; warmup = 2000; thin = 1;
##          total post-warmup draws = 8000
##
## Multilevel Hyperparameters:
## ~ID (Number of levels: 124)
##               Estimate Est.Error l-95% CI u-95% CI Rhat Bulk_ESS Tail_ESS
## sd(Intercept)     0.25      0.04     0.16     0.33 1.00     1851     2478
##
## Regression Coefficients:
##                                Estimate Est.Error l-95% CI u-95% CI Rhat
## Intercept                          1.10      0.19     0.72     1.48 1.00
## cultureSwiss                       0.91      0.15     0.62     1.20 1.00
## stimchild                          0.67      0.15     0.37     0.95 1.00
## siblings                           0.07      0.03     0.01     0.13 1.00
## stim_languagenative                0.03      0.07    -0.10     0.16 1.00
## months                             0.01      0.01    -0.01     0.03 1.00
## stimchild:siblings                -0.07      0.04    -0.14     0.00 1.00
## cultureSwiss:stimchild            -0.48      0.17    -0.81    -0.14 1.00
## cultureSwiss:siblings             -0.16      0.11    -0.37     0.05 1.00
## cultureSwiss:stimchild:siblings     0.11      0.12    -0.13     0.35 1.00
##                                Bulk_ESS Tail_ESS
## Intercept                          3997     5101
## cultureSwiss                       3349     4344
## stimchild                          3425     4979
## siblings                           3527     4343
## stim_languagenative                5773     5154
## months                             5610     5299
## stimchild:siblings                 3770     5654
## cultureSwiss:stimchild             3312     4856
## cultureSwiss:siblings              4872     5095
## cultureSwiss:stimchild:siblings     6518     5882
##
## Further Distributional Parameters:
##       Estimate Est.Error l-95% CI u-95% CI Rhat Bulk_ESS Tail_ESS
## sigma     0.38      0.03     0.34     0.43 1.00     2490     3641
##
## Draws were sampled using sampling(NUTS). For each parameter, Bulk_ESS
## and Tail_ESS are effective sample size measures, and Rhat is the potential
## scale reduction factor on split chains (at convergence, Rhat = 1).
```

Figure S4: Model summary of the multilevel Bayesian model analyzing infant looking times from study 1, excluding four participants from village 3. CI = credible interval.
