# Supplementary figures and images for: Input to the Language Learning Infant: The Impact of Other Children
Source: Dev Sci. 2025 Jul 20;28(5):e70045. doi: 10.1111/desc.70045 (PMC12277873; doi:10.1111/desc.70045)

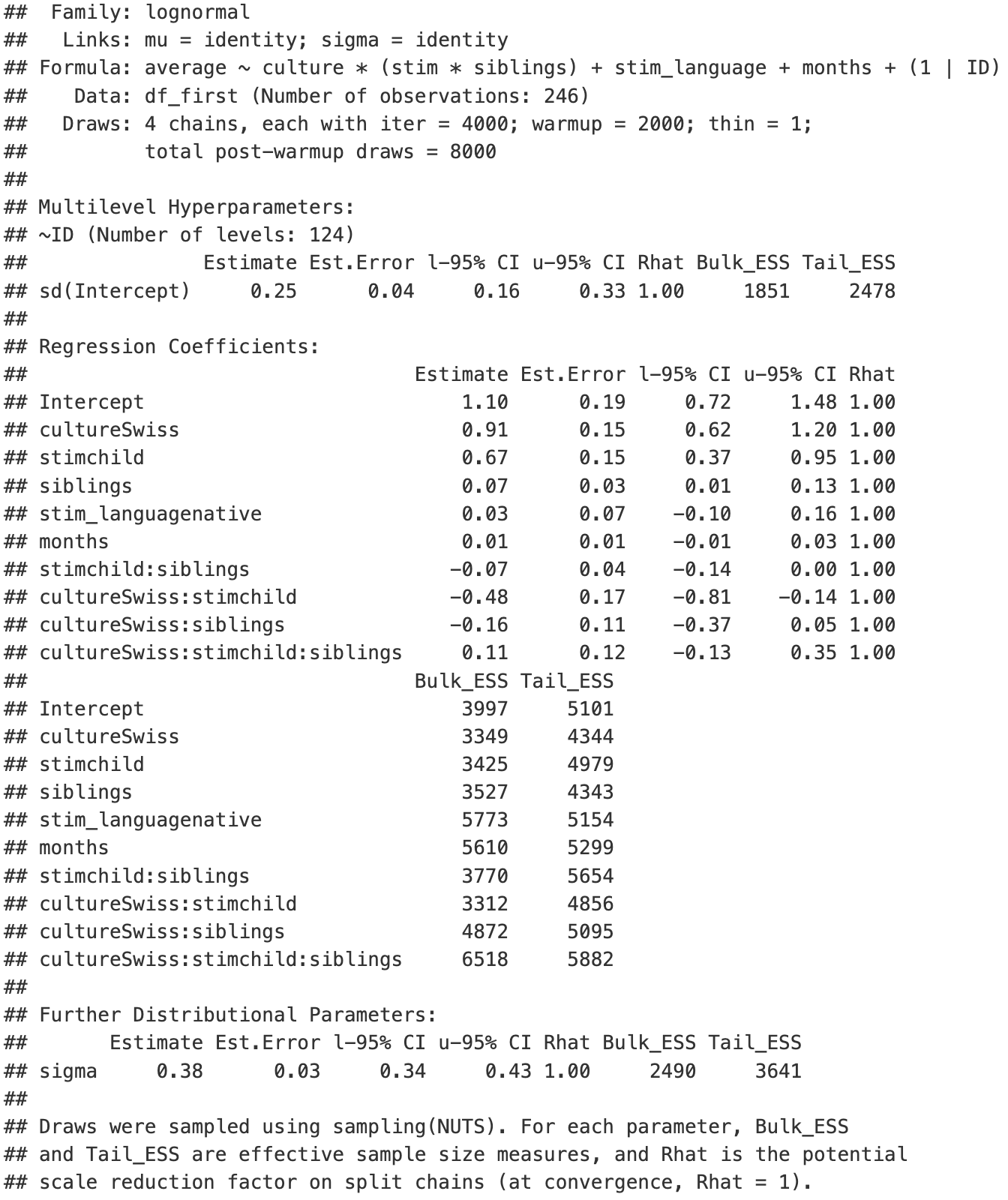

Supplement: Supplementary file 2 — desc70045‐sup‐0002‐SuppMat.zip [file DESC-28-e70045-s002.zip › suppl_materials_source_files/m3.png]

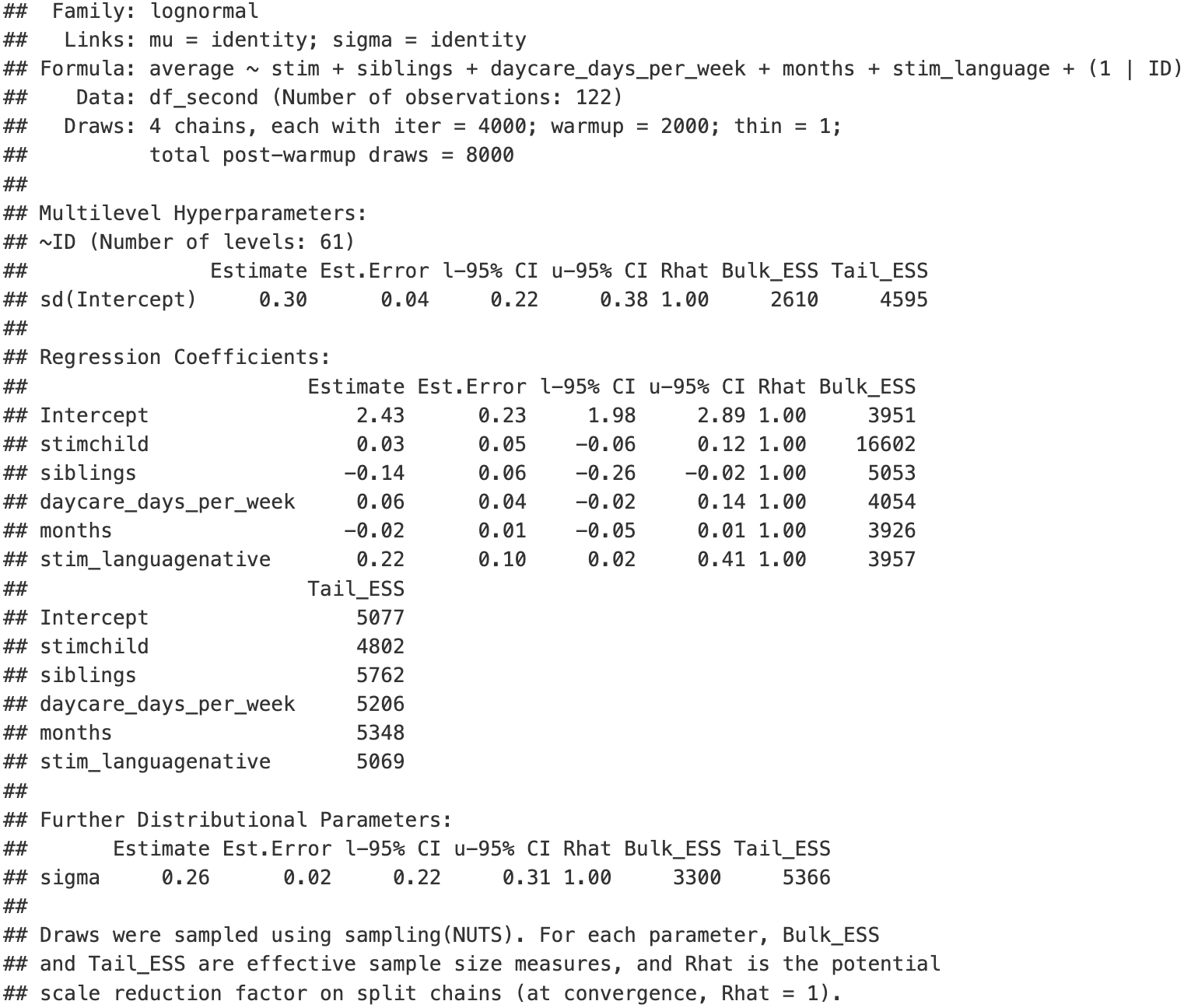

Supplement: Supplementary file 2 — desc70045‐sup‐0002‐SuppMat.zip [file DESC-28-e70045-s002.zip › suppl_materials_source_files/m2.png]

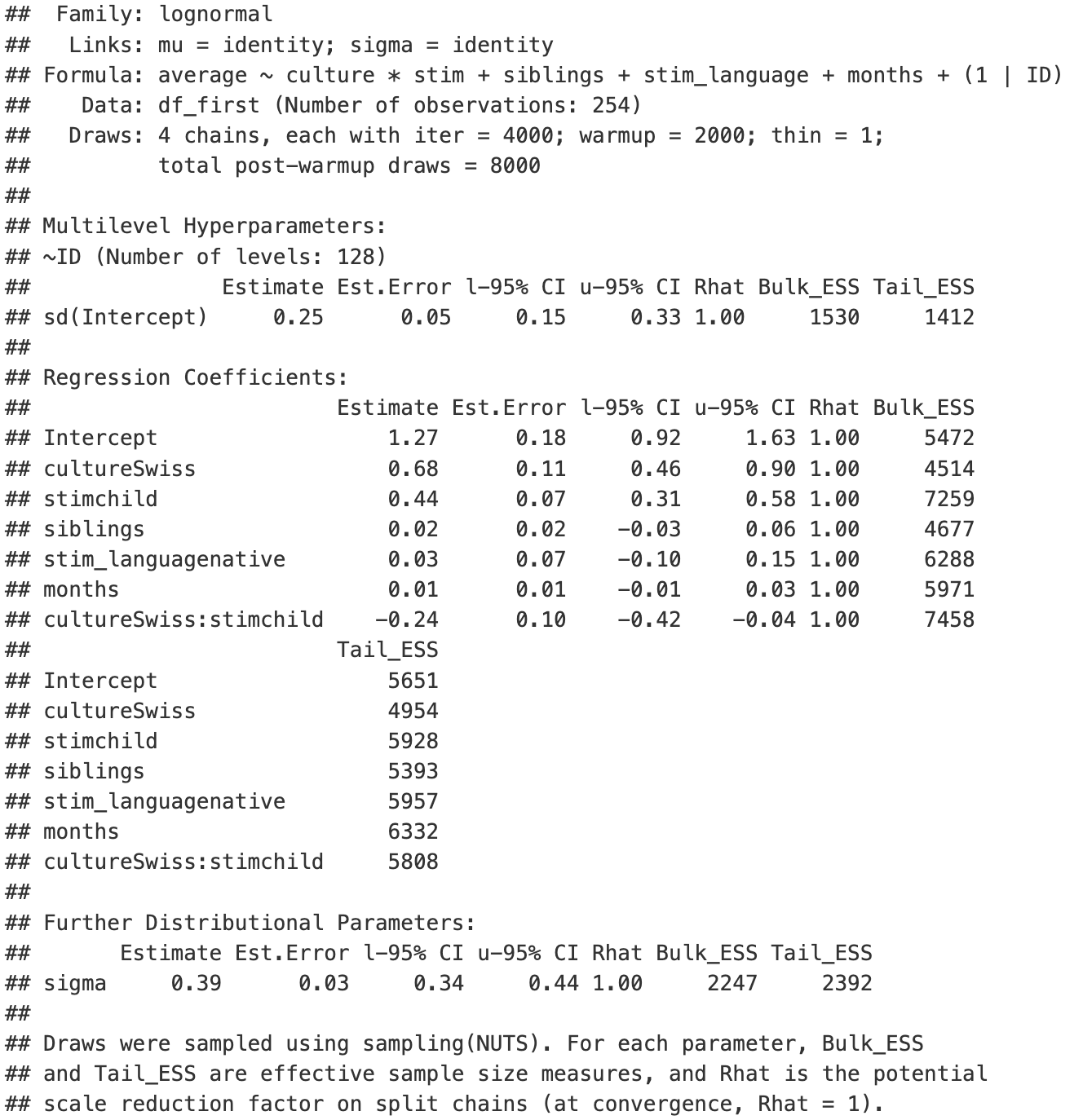

Supplement: Supplementary file 2 — desc70045‐sup‐0002‐SuppMat.zip [file DESC-28-e70045-s002.zip › suppl_materials_source_files/m1.png]

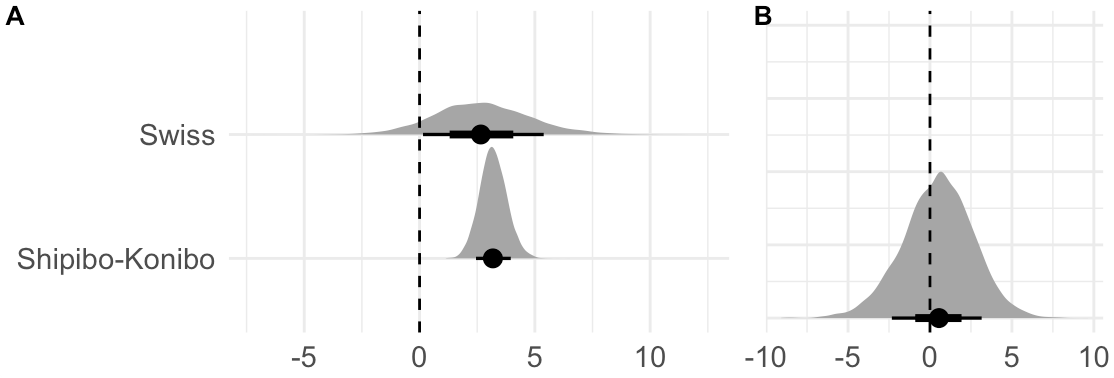

Supplement: Supplementary file 2 — desc70045‐sup‐0002‐SuppMat.zip [file DESC-28-e70045-s002.zip › suppl_materials_source_files/figure_S3.png]
